# Editorial: Non-precious metal-based materials: Design, fundamental and functionality

**DOI:** 10.3389/fchem.2022.1048038

**Published:** 2022-10-12

**Authors:** Xiaohang Zheng, Jinghuang Lin, Shude Liu, Xijun Wei

**Affiliations:** ^1^ School of Materials Science and Engineering, Harbin Institute of Technology, Harbin, China; ^2^ Institute of Engineering Innovation, The University of Tokyo, Tokyo, Japan; ^3^ International Center for Materials Nanoarchitectonics (WPI-MANA), National Institute for Materials Science, Tsukuba, Japan; ^4^ School of Mechanical Engineering, Yonsei University, Seoul, South Korea; ^5^ State Key Laboratory of Environment-Friendly Energy Materials, Tianfu Institute of Research and Innovation, School of Materials and Chemistry, Southwest University of Science and Technology, Mianyang, Sichuan, China

**Keywords:** non-precious, metal-based materials, function, energy, mechanism

Non-precious metal-based materials play a vital role in both functional and structural materials related to various fields of practical application. Non-precious metal-based materials show many advantages, such as diversity, low cost, rich valence and abundant reserves, which thus have gained more attention in the field of electrocatalysts. However, the limited electroactive sites, sluggish reaction kinetics and poor electrochemical stability of these electrocatalysts greatly limit their extensive application. Therefore, there is an urgent need to develop new-type non-precious metal-based materials as high-performance electrocatalysts. As a typical non-precious material, magnesium alloy is the lightest structural metal, which meets the requirements for energy saving and emission reduction. Understanding the ignition mechanism of magnesium alloys during casting processes may greatly enhance the processability and applications. However, the relationship between design, fundamentals, and functionality involving non-precious metal-based materials is less covered in the literature. Therefore, this present Research Topic aims to bring together the work that links the relationship between advanced non-precious metal-based materials with fine functionality, mainly focusing on 1) innovative, high-performance and dedicated materials for energy storage devices, 2) highly efficient and stable electrocatalysts for energy conversion devices, and 3) development of structural metal materials with high ignition resistance and anti-corrosion characteristics.

Nowadays, numerous non-precious metal-based materials have been developed as electrocatalysts for water splitting. For instance, Wang et al. reported Cu-based multicomponent metallic compound materials M-Cu (M = Mn, Fe, Co, Ni, and Pt) on copper foam substrate by a facile corrosion method, which was used as an electrocatalytic material for water splitting. As we know thatcopper foam is a large surface area support for a wide range of applications. Furthermore, the unusual physicochemical properties along with the multivalence band features of Cu ions are important for increasing activity towards catalytic reactions. In this case, the introduction of the second ions can change the valence states of copper and oxygen elements, resulting in a change in the electronic structure of the materials, thus changing its catalytic activity. This research suggested the important possibility of effectively hybridizing a second metal with an appropriate transition metal support to fine-tune the electrocatalytic activity and improve the cost-effectiveness of water splitting. Li et al. investigated the effect of bimetal element doping on Mn-based catalysts for selective catalytic reduction of NO_x_ with NH_3_. In the current work, a series of Zr and Co co-doped Mn-based oxides are developed by the coprecipitation method. The prepared Zr and Co co-doped Mn-based oxides exhibited greatly enhanced low-temperature denitrification performance, as well as good sulfur resistance and water resistance. Based on the analysis of various characterizations, the optimized Mn_6_Zr_0.3_Co_0.7_ catalyst showed the best performance, which mainly contributed to the large specific surface area and strong redox performance.

In addition, the ingenious regulation of synthetic process parameters is an important means to regulate the microstructure and optimize performance. Considering the important role of imine as intermediates for biological, agricultural and pharmaceutical applications, it is important to explore high-performance catalysts for synthesizing imine. For example, Chang et al. fabricated CeO_2_ with a fusiform structure by a combined microwave-ultrasonic approach. By changing the amount of H_2_O in the precursor system, the PVP micelle structures and resulting CeO_2_ surface coordination state were modified. The resulting CeO_2_ possessed high surface area and rich Ce^3+^ defects, which thus showed enhanced catalytic activity. The results confirmed that structural regulation and reaction mechanism are important for designing high-performance catalysts.

Magnesium alloys are the lightest structural metals, which meet requirements for energy saving and emission reduction. However, the ignition of magnesium alloys during casting processes limits their processability and applications. Zhao et al. systemically studied the ignition behavior of magnesium alloy melts based on experiments and calculations. The underlying ignition mechanism was revealed and the corresponding ignition critical conditions were proposed. This work is helpful to the development of new ignition-proof technologies for magnesium alloy casting processes, and provides valuable instructions for the high flame-retardant design of magnesium alloys.

In summary, this Research Topic discussed various non-precious metal-based materials for energy storage, energy conversion and structural applications, especially in understanding the relationship between design, fundamentals, and functionality. Owing to their key role in practical applications, engineering new high-performance non-precious metal-based materials is a vital research direction in the future.

